# *p*-Tolyl bis­(*p*-tolyl­amido)phosphate

**DOI:** 10.1107/S1600536809028281

**Published:** 2009-07-25

**Authors:** Mehrdad Pourayoubi, Saied Ghadimi, Ali Asghar Ebrahimi Valmoozi, Ali Reza Banan

**Affiliations:** a Department of Chemistry, Faculty of Sciences, Ferdowsi University of Mashhad, Mashhad 91779, Iran; b Department of Chemistry, Imam Hossein University, PO Box 16575-347, Tehran, Iran

## Abstract

In the title compound, C_21_H_23_N_2_O_2_P, the P atom exhibits tetra­hedral coordination; the P—N bond lengths are relatively short [1.6297 (13) and 1.6424 (13) Å]. In the crystal, adjacent mol­ecules are linked by N—H⋯O hydrogen bonds into a zigzag chain running along the *c* axis.

## Related literature

For related compounds, see: Pourayoubi & Sabbaghi (2007 ▶); Ghadimi *et al.* (2007 ▶); Gholivand *et al.* (2001 ▶). For bond-length data, see: Corbridge (1995 ▶).
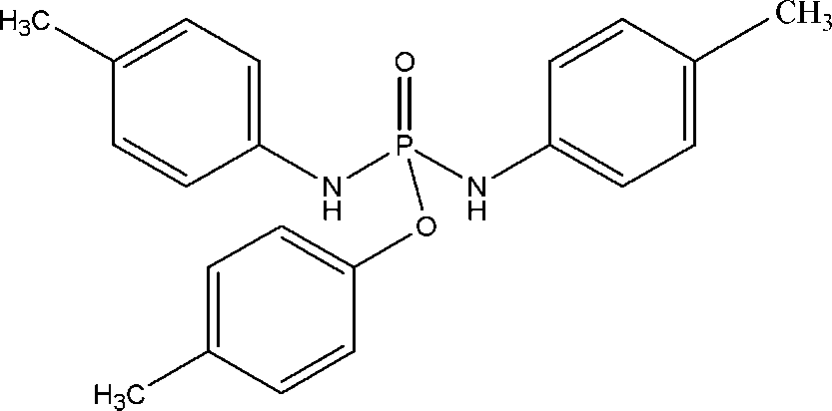


## Experimental

### 

#### Crystal data

C_21_H_23_N_2_O_2_P
*M* *_r_* = 366.38
Monoclinic, 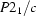

*a* = 14.0977 (6) Å
*b* = 14.7657 (6) Å
*c* = 9.5155 (4) Å
β = 104.676 (1)°
*V* = 1916.14 (14) Å^3^
*Z* = 4
Mo *K*α radiation
μ = 0.16 mm^−1^
*T* = 100 K
0.40 × 0.26 × 0.20 mm

#### Data collection

Bruker APEXII CCD area-detector diffractometer
Absorption correction: multi-scan (*SADABS*; Sheldrick, 1996 ▶) *T*_min_ = 0.939, *T*_max_ = 0.969
18737 measured reflections
5555 independent reflections
4484 reflections with *I* > 2σ(*I*)
*R*_int_ = 0.033

#### Refinement

*R*[*F* ^2^ > 2σ(*F* ^2^)] = 0.044
*wR*(*F* ^2^) = 0.107
*S* = 1.02
5555 reflections
243 parameters
2 restraints
H atoms treated by a mixture of independent and constrained refinement
Δρ_max_ = 0.62 e Å^−3^
Δρ_min_ = −0.50 e Å^−3^

### 

Data collection: *APEX2* (Bruker, 2005 ▶); cell refinement: *SAINT* (Bruker, 2005 ▶); data reduction: *SAINT*; program(s) used to solve structure: *SHELXS97* (Sheldrick, 2008 ▶); program(s) used to refine structure: *SHELXL97* (Sheldrick, 2008 ▶); molecular graphics: *SHELXTL* (Sheldrick, 2008 ▶); software used to prepare material for publication: *SHELXTL*.

## Supplementary Material

Crystal structure: contains datablocks I, global. DOI: 10.1107/S1600536809028281/ng2606sup1.cif

Structure factors: contains datablocks I. DOI: 10.1107/S1600536809028281/ng2606Isup2.hkl

Additional supplementary materials:  crystallographic information; 3D view; checkCIF report

## Figures and Tables

**Table 1:** Hydrogen-bond geometry (Å, °)

*D*—H⋯*A*	*D*—H	H⋯*A*	*D*⋯*A*	*D*—H⋯*A*
N1—H1*N*⋯O1^i^	0.878 (9)	1.928 (7)	2.805 (2)	176 (2)
N2—H2*N*⋯O2^ii^	0.881 (9)	2.209 (7)	3.068 (2)	165 (2)

Symmetry codes: (i) 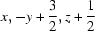; (ii) 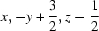.

**Fig. 1.**
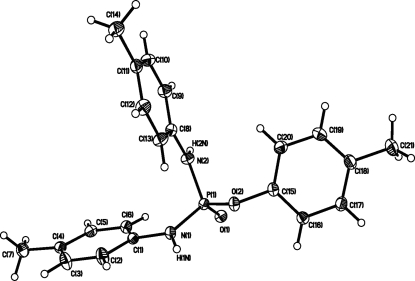
Molecular structure and atom-labeling scheme for [4-H3C—C6H4O]P(O)[NHC6H4-4-CH3]2 (50% probability ellipsoids).

**Fig. 2.**
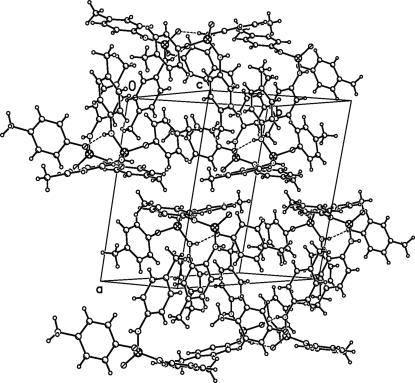
A view of unit cell packing of title compound.

## supplementary crystallographic information

### Comment

In the previous works about phosphoramidates and phosphoric acid esters, some derivatives have been structurally discussed such as [(CH_3_)_2_N][4-H_3_C—C_6_H_4_—O]P(O)CN (Ghadimi *et al.*, 2007), [(CH_3_)_2_N]P(O)[O—C_6_H_4_-(4-NO_2_)]_2_ (Gholivand *et al.*, 2001) and [(C_6_H_5_CH_2_)(CH(CH_3_)_2_)NH_2_][CCl_3_C(O)NHP(O)(O)(OCH_3_)] (Pourayoubi & Sabbaghi, 2007). Here, synthesis and crystal structure of a new phosphoramido acid ester, [4-H_3_C—C_6_H_4_O]P(O)[NHC_6_H_4_-4-CH_3_]_2_, are reported. The title compound was synthesized from the reaction of (4-tolyl)-dichlorophosphate with an excess amount of *para*-toluidine (1:4 mole ratios). Single crystals were obtained from CHCl_3_/CH_3_CN at room temperature. Molecular structure of [4-H_3_C—C_6_H_4_O]P(O)[NHC_6_H_4_-4-CH_3_]_2_ is shown in Fig. 1. The phosphorus atom has distorted tetrahedral configuration. The bond angles around P atom are in the range of 96.93 (6)° [for the O(2)—P(1)—N(1) angle] to 119.08 (7)° [for the O(1)—P(1)—N(1) angle]. The oxygen atom of OC_6_H_4_-4-CH_3_ moiety has *sp*^2^ character (the C(15)—O(2)—P(1) angle is 119.58 (9)°, also the P(1)—O(2) bond length of 1.6072 (11) Å is smaller than the P—O single bond length. The P(1)—O(1) bond length (1.4765 (11) Å) is longer than the normal P===O bond length [1.45 Å for P(O)Cl_3_, (Corbridge, 1995)]. The P—N bond lengths in title compound are shorter than the P—N single bond length [1.77 Å for NaHPO_3_NH_2_, (Corbridge, 1995)]. Moreover, the nitrogen atoms have *sp*^2^ hybridization. Sum of the surrounding angles around N(1) and N(2) atoms are 359.7° & 360.0°, respectively. H-bonded chain of title compound is formed *via* two different types of N—H···O===P hydrogen bond. A view of unit cell packing showing the N—H···O===P hydrogen bond is given in Fig. 2.

### Experimental

To a solution of (4-tolyl)-dichlorophosphate (2.250 g, 10 mmol) in 15 ml dry acetonitrile, a solution of *para*-toluidine (4.286 g, 40 mmol) in 30 ml acetonitrile was added at 0°C. After 4 h stirring, the solvent was evaporated in vacuum. The solid was washed with distilled water. Single crystals of the product were obtained from a solution of chloroform-acetonitrile (4:1) after a slow evaporation at room temperature. ^1^H NMR (250.13 MHz, CDCl_3_, 25°C, TMS), δ (p.p.m.): 2.17 (s, 6H, *p*-CH_3_), 2.25 (s, 3H, *p*-CH_3_), 6.95–7.00 (m, 8H, Ar—H), 7.03–7.15 (m, 4H, Ar—H), 8.15 (d, ^2^J(PNH) = 9.8 Hz, 2H, NH); ^13^C NMR (62.90 MHz, CDCl_3_, 25°C, TMS), δ (p.p.m.): 20.69 (s, 2 C, *p*-CH_3_), 2.77 (s, 1 C, *p*-CH_3_), 117.98 (d, ^3^J(P,*C*) = 7.6 Hz, C*_ortho_*), 120.77 (d, ^3^J(P,*C*) = 4.6 Hz, C*_ortho_*), 129.81 (s), 129.96 (s), 130.47 (s), 134.24 (s), 138.70 (d, ^2^J(P,*C*) = 1.8 Hz, 2 C, C*_ipso_*), 148.59 (d, ^2^J(P,*C*) = 6.5 Hz, 1 C, C*_ipso_*); ^31^P{^1^H} NMR (101.25 MHz, CDCl_3_, 25°C, H_3_PO_4_ external), δ (p.p.m.): 1.23 (s); ^31^P NMR, δ (p.p.m.): 1.23 (t, ^2^J(HNP) = 9.8 Hz). IR (KBr, cm^-1^): 3220 (NH), 2950, 2935, 1610, 1515, 1430, 1320, 1240 (P===O), 1190, 1150, 1125, 975, 915, 725.

### Refinement

The hydrogen atoms of NH groups were found in difference Fourier synthesis and refined in isotropic approximation with a distance restraint (*DFIX* 0.88 0.01). The H(C) atom positions were calculated. H atoms were refined in isotropic approximatiom in riding model with the *U*_iso_(H) parameters equal to 1.2 *U*_eq_(Ni), 1.2 *U*_eq_(Ci) or 1.5 *U*_eq_(Cii), where U(Ci) and U(Cii) are respectively the equivalent thermal parameters of nitrogen and carbon atoms of CH and CH_3_ groups to which corresponding H atoms are bonded.

### Crystal data

**Table:**

C_21_H_23_N_2_O_2_P	*F*(000) = 776
*M**_r_* = 366.38	*D*_x_ = 1.270 Mg m^−^^3^*D*_m_ = 0 Mg m^−^^3^*D*_m_ measured by not measured
Monoclinic, *P*2_1_/*c*	Mo *K*α radiation, λ = 0.71073 Å
Hall symbol: -P 2ybc	Cell parameters from 552 reflections
*a* = 14.0977 (6) Å	θ = 3–30°
*b* = 14.7657 (6) Å	µ = 0.16 mm^−^^1^
*c* = 9.5155 (4) Å	*T* = 100 K
β = 104.676 (1)°	Needle, colorless
*V* = 1916.14 (14) Å^3^	0.40 × 0.26 × 0.20 mm
*Z* = 4	

### Data collection

**Table:**

Bruker APEXII CCD area-detector diffractometer	5555 independent reflections
Radiation source: fine-focus sealed tube	4484 reflections with *I* > 2σ(*I*)
graphite	*R*_int_ = 0.033
ω scans	θ_max_ = 30.0°, θ_min_ = 1.5°
Absorption correction: multi-scan (*SADABS*; Sheldrick, 1996)	*h* = −19→19
*T*_min_ = 0.939, *T*_max_ = 0.969	*k* = −20→16
18737 measured reflections	*l* = −13→13

### Refinement

**Table:**

Refinement on *F*^2^	Primary atom site location: structure-invariant direct methods
Least-squares matrix: full	Secondary atom site location: difference Fourier map
*R*[*F*^2^ > 2σ(*F*^2^)] = 0.044	Hydrogen site location: inferred from neighbouring sites
*wR*(*F*^2^) = 0.107	H atoms treated by a mixture of independent and constrained refinement
*S* = 1.02	*w* = 1/[σ^2^(*F*_o_^2^) + (0.0306*P*)^2^ + 1.982*P*] where *P* = (*F*_o_^2^ + 2*F*_c_^2^)/3
5555 reflections	(Δ/σ)_max_ < 0.001
243 parameters	Δρ_max_ = 0.62 e Å^−^^3^
2 restraints	Δρ_min_ = −0.50 e Å^−^^3^

### Special details

**Table:**

Geometry. All e.s.d.'s (except the e.s.d. in the dihedral angle between two l.s. planes) are estimated using the full covariance matrix. The cell e.s.d.'s are taken into account individually in the estimation of e.s.d.'s in distances, angles and torsion angles; correlations between e.s.d.'s in cell parameters are only used when they are defined by crystal symmetry. An approximate (isotropic) treatment of cell e.s.d.'s is used for estimating e.s.d.'s involving l.s. planes.
Refinement. Refinement of *F*^2^ against ALL reflections. The weighted *R*-factor *wR* and goodness of fit *S* are based on *F*^2^, conventional *R*-factors *R* are based on *F*, with *F* set to zero for negative *F*^2^. The threshold expression of *F*^2^ > σ(*F*^2^) is used only for calculating *R*-factors(gt) *etc*. and is not relevant to the choice of reflections for refinement. *R*-factors based on *F*^2^ are statistically about twice as large as those based on *F*, and *R*- factors based on ALL data will be even larger.

### Fractional atomic coordinates and isotropic or equivalent isotropic displacement parameters (Å^2^)

**Table:**

	*x*	*y*	*z*	*U*_iso_*/*U*_eq_	
P1	0.30676 (3)	0.73955 (3)	0.60541 (4)	0.01332 (9)	
O1	0.37549 (8)	0.77353 (7)	0.52354 (11)	0.0174 (2)	
O2	0.27068 (8)	0.81531 (7)	0.70157 (11)	0.0165 (2)	
N1	0.34582 (9)	0.66666 (8)	0.73586 (13)	0.0154 (2)	
H1N	0.3574 (14)	0.6865 (13)	0.8256 (12)	0.020 (5)*	
N2	0.20970 (9)	0.69923 (9)	0.48821 (13)	0.0164 (2)	
H2N	0.2157 (13)	0.6978 (12)	0.3983 (11)	0.014 (4)*	
C1	0.37149 (10)	0.57578 (10)	0.71581 (15)	0.0153 (3)	
C2	0.38112 (14)	0.51526 (11)	0.83062 (17)	0.0249 (3)	
H2A	0.3725	0.5353	0.9192	0.030*	
C3	0.40352 (14)	0.42486 (12)	0.81343 (19)	0.0269 (4)	
H3A	0.4104	0.3853	0.8914	0.032*	
C4	0.41580 (11)	0.39238 (11)	0.68168 (18)	0.0198 (3)	
C5	0.40809 (11)	0.45397 (11)	0.56929 (17)	0.0189 (3)	
H5A	0.4170	0.4339	0.4809	0.023*	
C6	0.38729 (11)	0.54520 (10)	0.58541 (16)	0.0173 (3)	
H6A	0.3840	0.5854	0.5091	0.021*	
C7	0.43533 (13)	0.29353 (12)	0.6595 (2)	0.0277 (4)	
H7A	0.4415	0.2845	0.5623	0.042*	
H7B	0.3819	0.2578	0.6745	0.042*	
H7C	0.4950	0.2755	0.7276	0.042*	
C8	0.11965 (11)	0.66800 (10)	0.51075 (16)	0.0163 (3)	
C9	0.04290 (12)	0.64951 (11)	0.38934 (16)	0.0209 (3)	
H9A	0.0517	0.6582	0.2967	0.025*	
C10	−0.04647 (12)	0.61819 (12)	0.40582 (18)	0.0227 (3)	
H10A	−0.0964	0.6052	0.3237	0.027*	
C11	−0.06289 (11)	0.60580 (11)	0.54274 (18)	0.0201 (3)	
C12	0.01388 (12)	0.62506 (12)	0.66285 (17)	0.0222 (3)	
H12A	0.0044	0.6177	0.7554	0.027*	
C13	0.10467 (12)	0.65499 (11)	0.64861 (16)	0.0206 (3)	
H13A	0.1552	0.6663	0.7309	0.025*	
C14	−0.16075 (12)	0.57270 (13)	0.5597 (2)	0.0273 (4)	
H14A	−0.1587	0.5683	0.6610	0.041*	
H14B	−0.1746	0.5142	0.5151	0.041*	
H14C	−0.2112	0.6146	0.5136	0.041*	
C15	0.24805 (11)	0.90316 (10)	0.64444 (15)	0.0160 (3)	
C16	0.31733 (11)	0.97067 (10)	0.68618 (16)	0.0173 (3)	
H16A	0.3793	0.9576	0.7450	0.021*	
C17	0.29238 (12)	1.05864 (11)	0.63823 (17)	0.0206 (3)	
H17A	0.3381	1.1048	0.6667	0.025*	
C18	0.20005 (12)	1.07898 (11)	0.54811 (17)	0.0227 (3)	
C19	0.13342 (12)	1.00877 (12)	0.50624 (18)	0.0249 (3)	
H19A	0.0722	1.0210	0.4446	0.030*	
C20	0.15618 (12)	0.92020 (11)	0.55447 (17)	0.0215 (3)	
H20A	0.1107	0.8738	0.5268	0.026*	
C21	0.17365 (16)	1.17477 (13)	0.4986 (2)	0.0355 (4)	
H21A	0.1079	1.1762	0.4380	0.053*	
H21B	0.2182	1.1962	0.4446	0.053*	
H21C	0.1781	1.2129	0.5818	0.053*	

### Atomic displacement parameters (Å^2^)

**Table:**

	*U*^11^	*U*^22^	*U*^33^	*U*^12^	*U*^13^	*U*^23^
P1	0.01640 (17)	0.01403 (17)	0.00997 (15)	−0.00111 (13)	0.00415 (12)	0.00019 (13)
O1	0.0192 (5)	0.0202 (5)	0.0135 (5)	−0.0029 (4)	0.0054 (4)	0.0016 (4)
O2	0.0236 (5)	0.0143 (5)	0.0129 (4)	0.0008 (4)	0.0068 (4)	0.0008 (4)
N1	0.0225 (6)	0.0140 (6)	0.0100 (5)	−0.0003 (5)	0.0046 (4)	−0.0003 (4)
N2	0.0165 (6)	0.0222 (6)	0.0108 (5)	−0.0028 (5)	0.0041 (4)	−0.0006 (5)
C1	0.0152 (6)	0.0152 (7)	0.0154 (6)	−0.0011 (5)	0.0034 (5)	−0.0001 (5)
C2	0.0386 (9)	0.0204 (8)	0.0178 (7)	0.0034 (7)	0.0111 (6)	0.0029 (6)
C3	0.0383 (9)	0.0196 (8)	0.0239 (8)	0.0049 (7)	0.0102 (7)	0.0074 (6)
C4	0.0139 (6)	0.0170 (7)	0.0272 (8)	0.0010 (5)	0.0026 (6)	−0.0011 (6)
C5	0.0166 (7)	0.0203 (7)	0.0203 (7)	−0.0004 (6)	0.0054 (5)	−0.0043 (6)
C6	0.0196 (7)	0.0174 (7)	0.0157 (6)	−0.0011 (6)	0.0061 (5)	0.0005 (5)
C7	0.0245 (8)	0.0183 (8)	0.0375 (9)	0.0046 (6)	0.0025 (7)	−0.0010 (7)
C8	0.0170 (7)	0.0156 (7)	0.0166 (6)	−0.0004 (5)	0.0049 (5)	−0.0004 (5)
C9	0.0220 (7)	0.0248 (8)	0.0152 (6)	−0.0006 (6)	0.0033 (6)	0.0003 (6)
C10	0.0181 (7)	0.0253 (8)	0.0224 (7)	−0.0016 (6)	0.0010 (6)	−0.0010 (6)
C11	0.0178 (7)	0.0162 (7)	0.0269 (8)	0.0005 (6)	0.0068 (6)	0.0012 (6)
C12	0.0234 (7)	0.0255 (8)	0.0202 (7)	−0.0026 (6)	0.0100 (6)	0.0015 (6)
C13	0.0201 (7)	0.0259 (8)	0.0158 (6)	−0.0056 (6)	0.0044 (5)	−0.0013 (6)
C14	0.0192 (7)	0.0265 (9)	0.0369 (9)	−0.0019 (6)	0.0084 (7)	0.0020 (7)
C15	0.0213 (7)	0.0145 (7)	0.0131 (6)	0.0012 (5)	0.0063 (5)	0.0005 (5)
C16	0.0187 (7)	0.0181 (7)	0.0151 (6)	0.0017 (6)	0.0039 (5)	0.0000 (5)
C17	0.0248 (8)	0.0163 (7)	0.0200 (7)	−0.0015 (6)	0.0045 (6)	−0.0008 (6)
C18	0.0270 (8)	0.0186 (7)	0.0212 (7)	0.0046 (6)	0.0035 (6)	0.0016 (6)
C19	0.0218 (7)	0.0249 (8)	0.0244 (8)	0.0044 (6)	−0.0008 (6)	0.0032 (6)
C20	0.0206 (7)	0.0212 (8)	0.0209 (7)	−0.0025 (6)	0.0019 (6)	0.0004 (6)
C21	0.0402 (11)	0.0207 (9)	0.0380 (10)	0.0062 (8)	−0.0041 (8)	0.0041 (7)

### Geometric parameters (Å, °)

**Table:**

P1—O1	1.4765 (11)	C9—H9A	0.9300
P1—O2	1.6072 (11)	C10—C11	1.392 (2)
P1—N1	1.6297 (13)	C10—H10A	0.9300
P1—N2	1.6424 (13)	C11—C12	1.390 (2)
O2—C15	1.4114 (17)	C11—C14	1.510 (2)
N1—C1	1.4153 (19)	C12—C13	1.393 (2)
N1—H1N	0.878 (9)	C12—H12A	0.9300
N2—C8	1.4171 (19)	C13—H13A	0.9300
N2—H2N	0.881 (9)	C14—H14A	0.9600
C1—C6	1.390 (2)	C14—H14B	0.9600
C1—C2	1.391 (2)	C14—H14C	0.9600
C2—C3	1.391 (2)	C15—C16	1.382 (2)
C2—H2A	0.9300	C15—C20	1.383 (2)
C3—C4	1.393 (2)	C16—C17	1.392 (2)
C3—H3A	0.9300	C16—H16A	0.9300
C4—C5	1.387 (2)	C17—C18	1.398 (2)
C4—C7	1.510 (2)	C17—H17A	0.9300
C5—C6	1.395 (2)	C18—C19	1.388 (2)
C5—H5A	0.9300	C18—C21	1.507 (2)
C6—H6A	0.9300	C19—C20	1.396 (2)
C7—H7A	0.9600	C19—H19A	0.9300
C7—H7B	0.9600	C20—H20A	0.9300
C7—H7C	0.9600	C21—H21A	0.9600
C8—C13	1.394 (2)	C21—H21B	0.9600
C8—C9	1.395 (2)	C21—H21C	0.9600
C9—C10	1.388 (2)		
			
O1—P1—O2	114.12 (6)	C9—C10—C11	121.37 (15)
O1—P1—N1	119.08 (7)	C9—C10—H10A	119.3
O2—P1—N1	96.93 (6)	C11—C10—H10A	119.3
O1—P1—N2	108.07 (6)	C12—C11—C10	117.60 (14)
O2—P1—N2	108.03 (6)	C12—C11—C14	121.38 (15)
N1—P1—N2	109.90 (7)	C10—C11—C14	121.02 (15)
C15—O2—P1	119.58 (9)	C11—C12—C13	121.89 (14)
C1—N1—P1	124.90 (10)	C11—C12—H12A	119.1
C1—N1—H1N	117.2 (13)	C13—C12—H12A	119.1
P1—N1—H1N	117.6 (13)	C12—C13—C8	119.83 (14)
C8—N2—P1	129.74 (10)	C12—C13—H13A	120.1
C8—N2—H2N	116.7 (12)	C8—C13—H13A	120.1
P1—N2—H2N	113.6 (12)	C11—C14—H14A	109.5
C6—C1—C2	119.10 (14)	C11—C14—H14B	109.5
C6—C1—N1	122.17 (13)	H14A—C14—H14B	109.5
C2—C1—N1	118.73 (13)	C11—C14—H14C	109.5
C3—C2—C1	120.27 (15)	H14A—C14—H14C	109.5
C3—C2—H2A	119.9	H14B—C14—H14C	109.5
C1—C2—H2A	119.9	C16—C15—C20	121.99 (14)
C2—C3—C4	121.32 (15)	C16—C15—O2	118.56 (13)
C2—C3—H3A	119.3	C20—C15—O2	119.37 (13)
C4—C3—H3A	119.3	C15—C16—C17	118.52 (14)
C5—C4—C3	117.69 (15)	C15—C16—H16A	120.7
C5—C4—C7	120.50 (15)	C17—C16—H16A	120.7
C3—C4—C7	121.80 (15)	C16—C17—C18	121.35 (15)
C4—C5—C6	121.72 (14)	C16—C17—H17A	119.3
C4—C5—H5A	119.1	C18—C17—H17A	119.3
C6—C5—H5A	119.1	C19—C18—C17	118.22 (15)
C1—C6—C5	119.82 (14)	C19—C18—C21	121.04 (15)
C1—C6—H6A	120.1	C17—C18—C21	120.74 (16)
C5—C6—H6A	120.1	C18—C19—C20	121.53 (15)
C4—C7—H7A	109.5	C18—C19—H19A	119.2
C4—C7—H7B	109.5	C20—C19—H19A	119.2
H7A—C7—H7B	109.5	C15—C20—C19	118.37 (15)
C4—C7—H7C	109.5	C15—C20—H20A	120.8
H7A—C7—H7C	109.5	C19—C20—H20A	120.8
H7B—C7—H7C	109.5	C18—C21—H21A	109.5
C13—C8—C9	118.80 (14)	C18—C21—H21B	109.5
C13—C8—N2	122.85 (13)	H21A—C21—H21B	109.5
C9—C8—N2	118.35 (13)	C18—C21—H21C	109.5
C10—C9—C8	120.49 (14)	H21A—C21—H21C	109.5
C10—C9—H9A	119.8	H21B—C21—H21C	109.5
C8—C9—H9A	119.8		
			
O1—P1—O2—C15	40.60 (12)	C13—C8—C9—C10	−0.4 (2)
N1—P1—O2—C15	166.81 (11)	N2—C8—C9—C10	179.56 (15)
N2—P1—O2—C15	−79.60 (11)	C8—C9—C10—C11	1.1 (3)
O1—P1—N1—C1	−69.46 (14)	C9—C10—C11—C12	−0.6 (2)
O2—P1—N1—C1	167.95 (12)	C9—C10—C11—C14	179.31 (16)
N2—P1—N1—C1	55.89 (14)	C10—C11—C12—C13	−0.5 (2)
O1—P1—N2—C8	−172.64 (13)	C14—C11—C12—C13	179.52 (16)
O2—P1—N2—C8	−48.71 (15)	C11—C12—C13—C8	1.2 (3)
N1—P1—N2—C8	55.93 (15)	C9—C8—C13—C12	−0.7 (2)
P1—N1—C1—C6	15.1 (2)	N2—C8—C13—C12	179.30 (15)
P1—N1—C1—C2	−164.86 (13)	P1—O2—C15—C16	−99.59 (14)
C6—C1—C2—C3	−1.8 (3)	P1—O2—C15—C20	83.48 (16)
N1—C1—C2—C3	178.16 (16)	C20—C15—C16—C17	1.4 (2)
C1—C2—C3—C4	−0.7 (3)	O2—C15—C16—C17	−175.40 (13)
C2—C3—C4—C5	2.1 (3)	C15—C16—C17—C18	−0.9 (2)
C2—C3—C4—C7	−177.02 (17)	C16—C17—C18—C19	−0.5 (2)
C3—C4—C5—C6	−1.0 (2)	C16—C17—C18—C21	179.21 (16)
C7—C4—C5—C6	178.12 (15)	C17—C18—C19—C20	1.4 (3)
C2—C1—C6—C5	2.9 (2)	C21—C18—C19—C20	−178.31 (17)
N1—C1—C6—C5	−177.10 (14)	C16—C15—C20—C19	−0.6 (2)
C4—C5—C6—C1	−1.5 (2)	O2—C15—C20—C19	176.23 (14)
P1—N2—C8—C13	−9.5 (2)	C18—C19—C20—C15	−0.9 (3)
P1—N2—C8—C9	170.57 (12)		

### Hydrogen-bond geometry (Å, °)

**Table:**

*D*—H···*A*	*D*—H	H···*A*	*D*···*A*	*D*—H···*A*
N1—H1N···O1^i^	0.88 (1)	1.93 (1)	2.805 (2)	176 (2)
N2—H2N···O2^ii^	0.88 (1)	2.21 (1)	3.068 (2)	165 (2)

Symmetry codes: (i) *x*, −*y*+3/2, *z*+1/2; (ii) *x*, −*y*+3/2, *z*−1/2.
